# PANCREATIC PANNICULITIS IN A CHILD

**DOI:** 10.4103/0019-5154.62756

**Published:** 2010

**Authors:** Angoori Gnaneshwar Rao, Indira Danturty

**Affiliations:** *From the Department of Dermatology, Gandhi Medical College, India.*

**Keywords:** *Ghost cells*, *panniculitis*, *pseudopancreatic cyst amylase*

## Abstract

Pancreatic panniculitis is an uncommon cause of panniculitis. It is associated with acute or chronic pancreatic disease. 1½ year old boy was referred for erythematous tender nodules involving both legs and associated with abdominal distention, fever, and vomiting. Dermatological examination revealed multiple erythematous tender nodules distributed over front and back of legs. Blood chemistry showed raised serum amylase (430 IU/ 1). Ultrasonography showed a pseudopancreatic cyst. Multislice CT scan was suggestive of a pseudopancreatic cyst. A skin biopsy showed typical features of pancreatic panniculitis which included lobular panniculitis with lipocyte degeneration with few Ghost cells.

## Introduction

Pancreatic panniculitis is an uncommon cause of panniculitis and often presents as purple-red tender nodules or plaques on the extremities, trunk, and buttocks. These lesions later may become fluctuant and spontaneously ulcerate, discharging oily brown exudates.[1] It is associated with acute or chronic pancreatic disease. It may be associated with arthritis, pleural effusion, pericardial effusion, GI bleeding, ascites, and mesenteric thrombosis.

## Case Report

**Figure 1:**
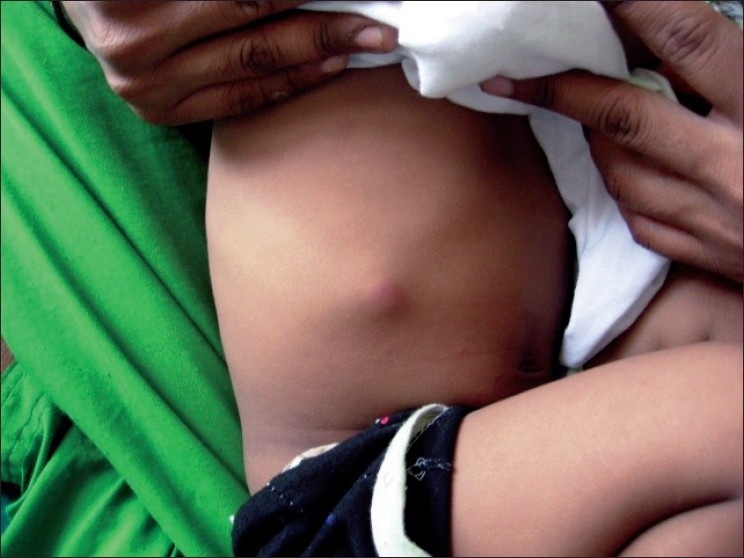
Erythematous nodule on the right costal margin

**Figure 2:**
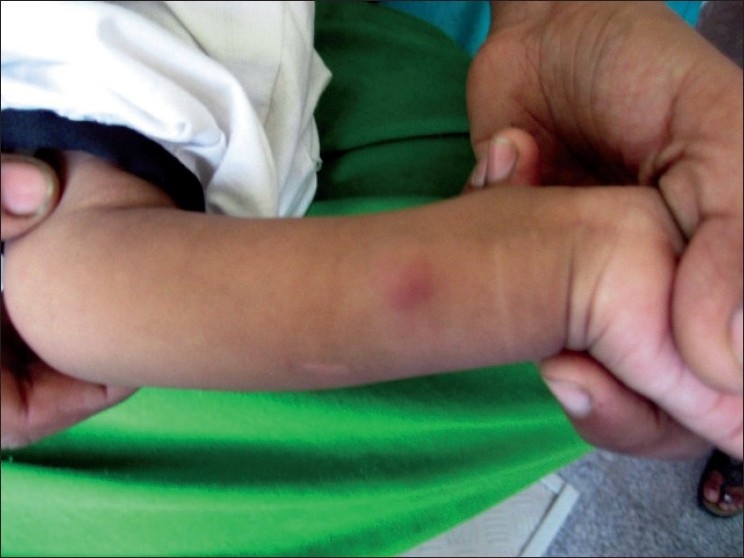
Erythematous nodule on flexor aspect of the left wrist

**Figure 3:**
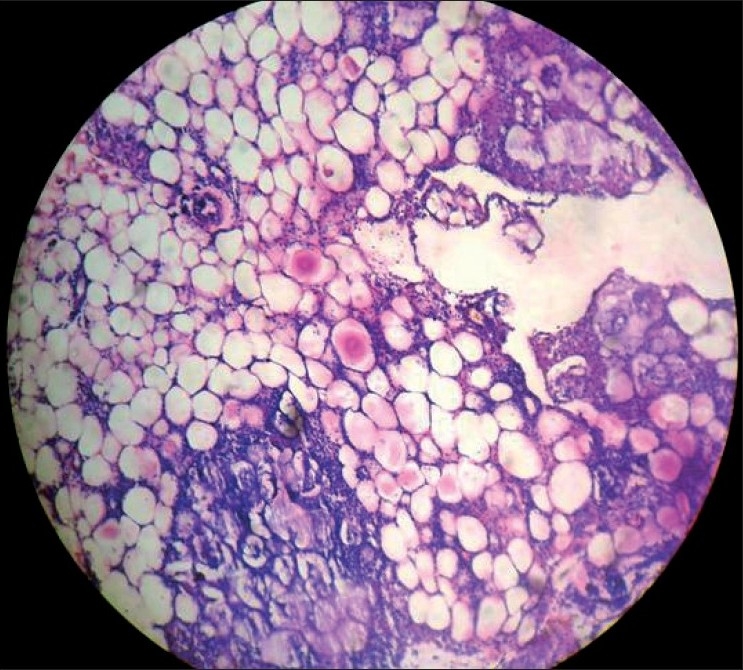
HPE from the nodule showing lobular panniculitis with focal areas of lipocyte degeneration (H and E stain, magnification × 100)

**Figure 4:**
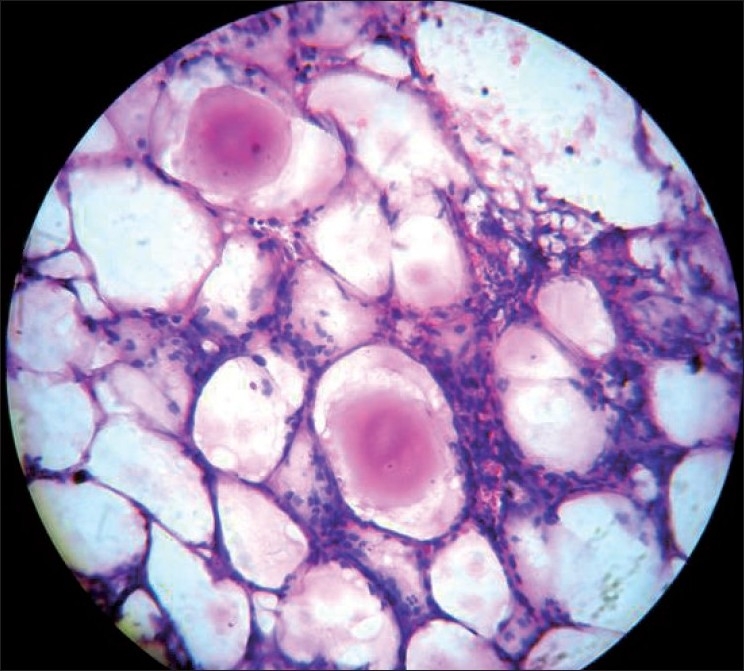
HPE from the nodule showing Ghost cells (H and E stain, magnification × 400)

2½ year old boy was referred to dermatology department for multiple cutaneous nodules involving both legs of 5 days duration. The child was admitted with abdominal distention associated with fever and vomiting. He was provisionally diagnosed with intestinal obstruction. General examination revealed sacral and bilateral pedal edema. Cutaneous examination revealed multiple erythematous tender nodules distributed over front and back of legs, and front of thighs. Routine investigations (CBP, complete urine examination, [Figures 1 and 2] blood sugar, blood urea, serum creatinine, liver function tests, X-ray chest) were normal. HIV 1 and 2 were non reactive. Serum amylase was raised 430 IU/l (normal: 60 to 180 IU/l). Ascitic fluid amylase level was 2080 IU/l. Ultrasonography revealed pseudopancreatic cyst. Multislice CT scan was suggestive of psedopancreatic cyst. A skin biopsy taken from nodule showed lobular panniculitis with focal areas of lipocyte degeneration with few ghost cells. Inflammatory infiltrate in the lobule consists of lymphocytes, macrophages, and polymorphs [Figures 3 and 4].

## Discussion

Pancreatic panniculitis was first described by Hans Chiari in 1883, affecting 2% to 3% of patients with pancreatic diseases.[2] It is associated with acute or chronic pancreatitis, pancreatic trauma, pancreatic adenocarcinoma, pancreatic neuroendocrine tumor, pancreatic divisum, systemic lupus erythematosus, human immunedeficiency virus infection, and hemophagocytic syndrome. Pancreatic carcinoma and pancreatitis are most intimately associated with pancreatic panniculitis.[1] Specifically, acinic cell adenocarcinoma is responsible for more than 50% of all cases,[3] though only 16% of acinic cell adenocarcinoma present with panniculitis.[4] In addition to eosinophilia, leukemoid reaction, and hypocalcemia, patients may have systemic involvement including mesenteric thrombosis, ascites, and pleural and pericardial effusions.[5,6] Additional pathological changes accompany the skin lesions and relate to lypocyte degeneration in other organs. Periarticular lipocyte degeneration results in a secondary acute arthritis that most frequently involves the ankles and may be migratory, intermittent, or persistent. Other joints subsequently or concurrently may be involved, including the knees, metacarpals, wrists, and elbows. Arthritis has been reported in 54% to 88% of cases[1,7] More rarely submucosal lipocyte degeneration resulting in gastrointestinal tract bleeding can occur.[1] Common laboratory abnormalities associated with pancreatic panniculitis include elevated sedimentation rates and lipase and trypsin levels. A differential diagnosis of panniculitis that may resemble pancreatic panniculitis could include erythema nodosum; sclerosing panniculitis; a_1_-antitrypsin deficiency panniculitis; cutaneous polyarteritis nodosa; nodular vasculitis; lupus panniculitis; and infective, traumatic, and factitial panniculitis.[8–11]

The characteristic histopathological features of pancreatic panniculitis was first described by Szymanski and Bluefarb[12] in 1961. Infact, Ball and colleagues[13] have suggested that pancreatic panniculitis may begin as septal panniculitis and only later develop lobular involvement. Histopathology is pathognomonic consisting of lobular panniculitis highlighted by focal areas of lipocyte degeneration populated by anucleate necrotic adipocytes surrounded by thickened acidophilic cell membranes, termed ghost cells. There was a dense infiltration of lymphocytes, macrophages, neutrophils. A unique feature, when present, is the deposition of granular or homogenous basophilic material resulting from the saponification of fat by calcium salts.[8]

Although there is no universally accepted mechanism for the development of the skin lesions, a popular hypothesis states that a synergism exists between the elevated serum levels of lipase and trypsin. Trypsin alters the permeability of the tissue blood vessels, which allows lipase to hydrolyze lipids in the adipocyte cell membranes and interior, which leads to lipocyte degeneration of the tissue.[14,15]

Successful treatment of pancreatic panniculitis usually requires diagnosis and treatment of the underlying pancreatic pathology. As the pancreatic enzyme levels decrease, the skin lesions usually tend to regress.[7] There has been some success reported with the administration of octreotide acetate, a synthetic polypeptide that inhibits pancreatic enzyme production.[9,10,16] In addition, general supportive measures, including rest, elevation of the legs, compression stockings, and nonsteroidal anti-inflammatory drugs, may be helpful.

To our knowledge, our patient represents the youngest case of pancreatic panniculitis. Prior to this, Suwattee *et al.* have reported pancreatic panniculitis in 4 year old child with nephrotic syndrome.[17]
